# Development of a mobile application for assessing reaction time in walking and TUG duration: Concurrent validity in female older adults

**DOI:** 10.3389/fmed.2023.1076963

**Published:** 2023-02-01

**Authors:** Ampha Pumpho, Supapon Kaewsanmung, Petcharat Keawduangdee, Patcharawan Suwannarat, Rumpa Boonsinsukh

**Affiliations:** ^1^Department of Physical Therapy, School of Integrative Medicine, Mae Fah Luang University, Chiang Rai, Thailand; ^2^Department of Neurological Rehabilitation, Mae Fah Luang University Hospital, Chiang Rai, Thailand; ^3^Faculty of Physical Therapy, Srinakharinwirot University, Nakhon Nayok, Thailand

**Keywords:** concurrent validity, mobile, application, reaction time, walking

## Abstract

**Introduction:**

The TUG can be used to distinguish between people who fall and people who don’t fall. To evaluate cognitive dual-task performance while walking for fall prediction, TUG-dual was frequently employed. A recent study has created a mobile application that enables simple interaction to provide greater convenience for monitoring the duration of TUG, TUG-subtraction, and reaction time.

**Objective:**

The research aim was to ascertain the concurrent validity of the mobile application that was developed for the clinical assessment of TUG, TUG-subtraction, and reaction time.

**Methods:**

Twenty-nine older persons participated in this study. The testing protocol involved the TUG, TUG-subtraction, and reaction time assessment. For TUG and TUG-subtraction, the duration to complete the task was recorded by the APDM Mobility Lab system and the mobile application. For the reaction time tests, the reaction times (msec) were recorded by the Multi Choice Reaction timer and the Mobile application. The TUG durations recorded by the APDM Mobility Lab system were correlated with those recorded by the mobile application to verify the concurrent validity using Pearson’s product moment correlation coefficient. Also, the reaction time by the Multi Choice Reaction timer was correlated with the mobile application. Bland-Altman plots were used to explore the existence of any systematic differences between the measurements.

**Results:**

Our results showed very strong correlations between the TUG and TUG-subtraction duration derived from the APDM Mobility Lab system and the mobile application (*r* = 0.96 and 0.96, respectively). For the reaction time, the results showed a moderate correlation between the reaction time derived from the mobile application and the Multi Choice Reaction Timer (*r* = 0.67).

**Conclusion:**

The mobile application, which allows measurement in TUG and TUG-subtraction, is a highly valid tool for TUG duration assessment. However, this application is capable for assess the reaction time with moderate validity for reaction time assessment.

## Introduction

Executive function refers to a group of cognitive abilities that need for complex goal-directed activity to be planned, initiated, sequenced, and monitored (1). Measurement of an executive function yields valuable data for predicting falls (2). According to the dual-task methodology, a person should complete the task that is being assessed for its attention demand (primary task) while concurrently executing a different task (secondary task) (3). The Timed Up and Go test (TUG) is an easy and validated procedure to evaluate functional mobility and may also be useful to monitor clinical changes over time in older individuals (4). The TUG is a reliable assessment with excellent test-retest reliability in older adults (*ICC* > 0.98), and also in patients with Parkinson’s disease (*ICC* = 0.8) or patients undergoing hemodialysis (*ICC* = 0.96) (4–6). Utilizing TUG in combination with the cognition task of subtraction by three from a random number between 20 and 100, is known as the Timed Up and Go cognitive (TUG cognitive or TUG-subtraction) (7). During the past two decades, the TUG cognitive has been applied to measure cognitive-motor interference (CMI) during walking in various people (8). Gait deterioration during dual-task testing relative to single-task performance was associated with increased fall risks (9). For identifying older persons who live in the community and are at risk of falling, the TUG cognitive showed 80% sensitivity and 93% specificity (10). In the clinical setting, the administration of TUG-subtraction requires the clinician to work multi-tasking, including measuring the time to complete TUG activity, calculating the number of correct subtraction answers, and guarding the participant during TUG cognitive performance. Therefore, there is a need for a tool that could help the clinician to administer the TUG-subtraction, subsequently leading to the reduction of measurement error. Apart from subtraction, other cognitive tasks are also used for assessing dual-task performance. According to the classification system by Al-Yahya et al. (11), the cognitive task can be classified into five types, including working memory, mental tracking, reaction time, discrimination and decision-making, and verbal fluency. The reaction time test is a useful cognitive ability test that may further search for the cognitive processing components that may explain individual differences in psychometric intelligence (12). The number of stimuli in a task that needs to specific motor reaction can be used to categorize reaction time tasks. The reaction time task is known as a simple reaction time task if there is exactly one stimulus; if there is more than one, it is known as a choice reaction time task (13). In order to complete the necessary operation within a set amount of time, many cognitive operations require speedy sufficient information processing (14). Therefore, it is helpful to understand the nature of the related attention deficit to employ tests of response speed that directly evaluate processing speed (15). For instance, the impaired alertness system, which mostly involves the frontal regions of the brain, is linked to delayed reaction times (16). Reaction time tests are frequently used in computerized cognitive assessment; these are normally performed by displaying a stimulus on a computer monitor and asking the individual to respond as quickly as possible (using a keypad or a computer mouse) (17). However, the reaction time test has not been frequently performed in the clinical setting due to a lack of a standardized measurement tool. Moreover, most of the computerized assessments for reaction time are administered while seated in front of the computer, which limits its use when information about the reaction time during walking is required.

Nowadays, the use of smartphones and the internet has consistently increased markedly in Thailand (18). In Thailand, around 53.57 million people used smartphones in 2020, a significant rise from 2017, when just 42.15 million people used smartphones (18). In particular, mobile applications related to health and fitness are expanding (19). These applications may provide users convenience and are easily accessible for low or no expense. Recently, we developed the mobile application (Walking Think) that allows easy interaction with users to record the duration of a single task (TUG), dual-task walking with the cognitive task, and measurement of reaction during sitting and walking. “Walking Think” also provides past records for comparison purposes that enable the therapist to better plan the intervention accordingly. This application was created with Android Studio 4.2.1 (Google, Mountain View, CA). Also, it is freely available for download with permission. The aim of this study was to determine the concurrent validity of this newly developed mobile application (Walking Think) with the standard laboratory equipment (APDM’s Mobility Lab™ and Multi Choice Reaction Timer). In this study, the reaction time function of the Walking Think was validated in sitting due to the protocol of the reference test (Multi Choice Reaction Timer).

## Materials and methods

### Participants

The participants were recruited from the community based on the following inclusion criteria: (1) Age 60–80 years, (2) medically stable, and (3) able to walk independently without walking aids for at least 10 m. Subjects were excluded if they had (1) neurological disorders that sufficiently disturb the balance, (2) hearing loss, (3) severe visual impairment, (4) orthopedic conditions or pain affecting natural gait, and (5) comprehension issues, indicated by a score of less than 24 on the Thai version of the Mini-Mental State Examination (MMSE) (20).

The sample size calculation for Pearson’s product moment correlation coefficient was performed using G*power version 3.1.9.7. For this study, a minimum total of 29 subjects were needed, based on the estimated values of error probability (α) at 0.05 and 80% power.

### Measurement tools

Baseline information including age, gender, and underlying disease was collected from all participants by interviewing.

1. Mobile Application (Walking Think). We developed an Android-based mobile application running on version 7 or higher. The application was installed on a Huawei P30 (Huawei Technologies Co., Ltd., Guangdong, China). This application was designed to allow easy interaction with the user to record the walking assessment (e.g., walking test, TUG test, reaction time test, and dual-task assessment). This application achieves three main functionalities:
  1.1 Gait duration monitoring. The application was designed such that it could be utilized by either the clinician or the self-administered older adults to manually start and stop gait recording by pressing the start/stop button. To clarify the concurrent validity of the application to measure the duration of TUG and TUG-subtraction, the rater carried the phone and started the recording while observing the participants get up from a chair and stop when they were sitting down. The rater is a physiotherapist with more than 10 years of experience who has had extensive training in TUG assessment.
  1.2 Reaction time. The reaction time modality was designed to generate the vibration randomly every 2–4 s, which deactivates by manually pressing the screen for stop. The reaction time will be recorded in milliseconds. The administration of reaction time assessment *via* this application can be selected to assess either as a simple task (while seated or standing) or dual-task (while walking or performing the TUG test).
  1.3 Summary results display. The application stores the information about the previous assessment results and can display it in a graph, allowing the users to follow the progression.
2. APDM’s Mobility Lab™ (APDM Inc., Portland, OR, USA). The data was gathered and kept in the APDM Inc., Gait cycles and associated events were identified and calculated using a gyroscope (±400°/s range) and an accelerometer (±5 g range) to record angular movement and acceleration at a sampling rate of 200 Hz. On the participants, four portable initial sensors were positioned at the sternum, 5th lumbar vertebrae, and left and right foot. Each trial is recorded by APDM Inc., based on the configuration of the TUG plug-in.
3. Multi Choice Reaction Timer (Mahidol University) consisted of the following components: (1) A stimulus unit generating auditory stimulus 2,800 Hz, (2) a microswitch keyboard with a press button activated about 5 mm. movement of the finger, and (3) a digital watch counting the time between stimulus “on” and response “off” in 1/1,000 of a second.

### Task and procedures

The inertial sensors were placed on the participants when performing the TUG and TUG-subtraction. Participants received standardized verbal instruction about the procedures. After the practice trial, participants performed TUG followed by TUG-subtraction. Next, the sequence of reaction time tests by the Multi Choice Reaction Timer or reaction time test by the Walking Think application was randomized for each participant. The participants were asked to perform two trials of TUG or TUG-subtraction, but one trial of each reaction time test.

The TUG procedure required the participants to rise up from their chairs, walk 3 m at a self-selected speed, turn around, and walk back and seat down. For the TUG-subtraction, a random number between 20 and 100 was chosen to begin the serial subtraction of three, and participants were instructed to recite it aloud. The instruction for performing TUG-subtraction was “please do both tasks as well as you can.” There was no instruction to prioritize either gait or subtraction tasks. For the second trial of TUG-subtraction, different digit number, unlike those performed during the first trial, was given to avoid the learning effect. During the TUG performance, two raters scored at the same time: one rater used the APDM Inc., while another rater used the Walking Think application. Digital recorders were used to record participant responses to the subtraction task.

For reaction time assessment using the Multi Choice Reaction Timer, the participants were instructed to take a seat in front of a desk with their forearms resting on it comfortably. They were also instructed to place their index finger lightly on the reaction timer’s microswitch key and be prepared to press it as fast as they heard the auditory stimulus. For reaction time assessment using the mobile application, the participants were instructed to hold the mobile in one hand while sitting comfortably on a chair. After feeling the vibration stimulus on their same hand, participants were instructed to quickly push the screen with their thumb. The assessment of reaction time in each instrument was performed for 1 min.

### Data analyses

Descriptive statistics (mean and SD) were calculated for TUG duration or reaction time. Agreement between the TUG parameters from APDM and Walking Think, and reaction time between the Muli Choice Reaction Timer and Walking Think were utilized to evaluate Bland-Altman and limits of agreement (LoA), percentage error, and Pearson’s and concordance correlation coefficients. Pearson’s (*r*) and concordance correlation coefficients (*r*_*c*_) were used to analyze the relative and overall agreement between the two methods, respectively (21). Correlation thresholds were set as negligible (*r* ≤ 0.30), low (0.30 < *r* ≤ 0.50), moderate (0.5 < *r* ≤ 0.70), high (0.70 < *r* ≤ 0.90), and very high (0.90 < *r* ≤ 1.00) (22). Bland-Altman plots were used to explore the existence of any systematic differences between the measurement (23). The NCSS 2022 version 22.0.3 statistic software was used for all statistical analyses.

## Results

**FIGURE 1:**
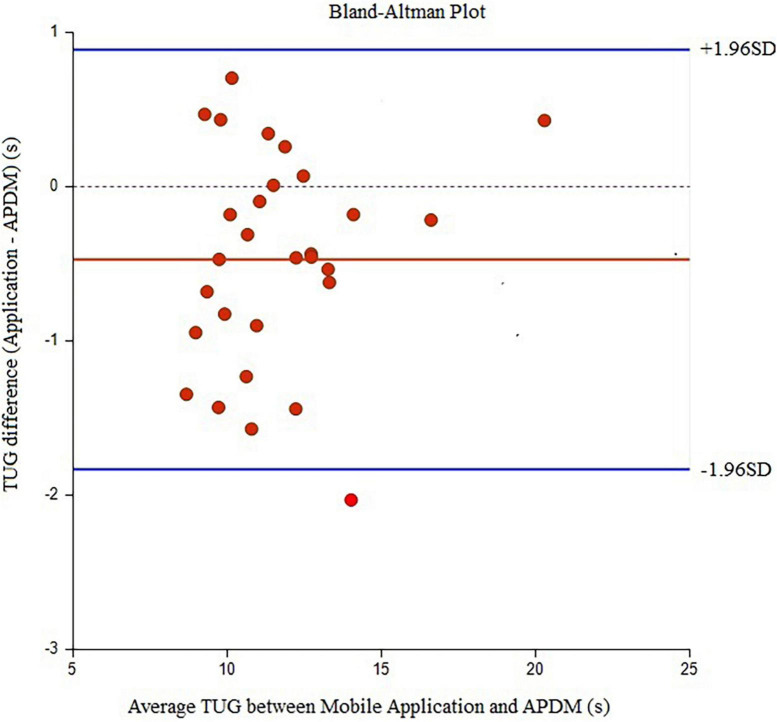
Bland-Alman graphs comparing the difference between the average values of TUG duration measured by the Walking Think mobile application and APDM.

**FIGURE 2:**
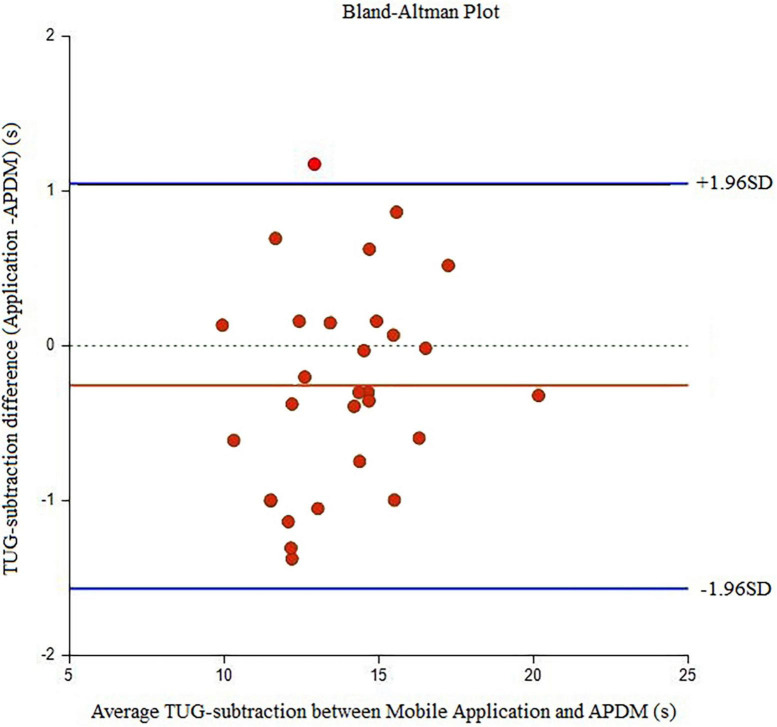
Bland-Alman graphs comparing the difference between the mean values of TUG cognitive duration measured by the Walking Think mobile application and APDM.

**FIGURE 3:**
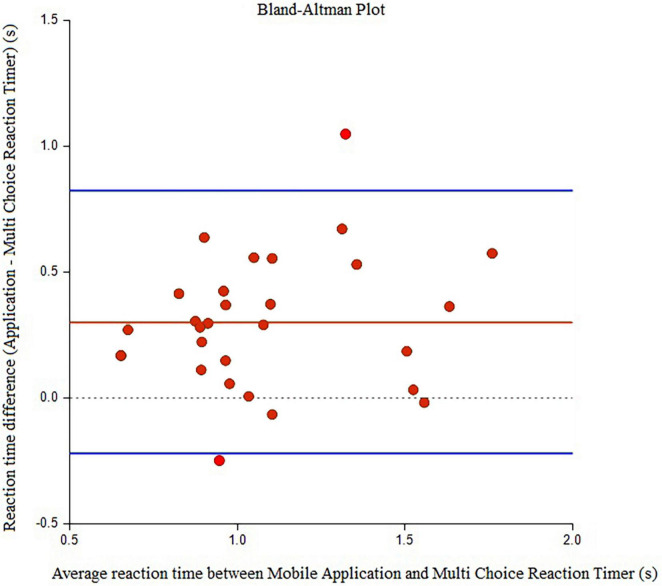
Bland-Alman graphs comparing the difference between the mean values of reaction time measured by the Walking Think mobile application and Multi Choice Reaction Timer.

Twenty-nine elder women (age: 65.90 ± 4.18 years, MMSE score: 26.47 ± 0.88) participated in the study. In relation to the history of falls, two participants (6.9%) reported falls in the past 6 months with no related injury. The underlying diseases of the participants and their medications were reported (Table 1). There were six individuals with one pathology (HT or DM or Asthma), four individuals with two pathologies (HT and DLP, or HT and DM), and five individuals with three pathologies (HT and DLP and DM). Table 2 displays the Mean (±SD) values for each outcome measure. In Tables 3, 4, agreement values are presented. TUG and TUG-subtraction duration had very high relative and overall agreement between devices (*r* and *r*_*c*_ values >0.9). The points on the Bland-Altman plot were uniformly and tightly scattered around the horizontal axis (Figures 1, 2). However, reaction time possessed moderate relative agreement and low overall agreement (*r* = 0.67, *r_*c*_* = 0.46). Also, the points on the Bland-Altman plot were widely spread and scattered around the horizontal axis (Figure 3).

**TABLE 1:** Distribution of the different diseases involved in the study.

Underlying diseases	Number of occurrences (total of 29 individuals)	Pharmacology
Hypertension	12	Amlopin
Dyslipidemia	8	Simvastatin
Diabetic mellitus	8	Metformin
Asthma	1	Albuterol

**TABLE 2:** Time Up and Go duration and reaction time using the Walking Think mobile application and other reference devices.

Variables	Walking Think	APDM	Multi choice reaction timer
TUG duration (s)	11.43 ± 2.54	11.90 ± 2.43	–
TUG-subtraction duration (s)	13.70 ± 2.31	13.96 ± 2.17	–
Reaction time (s)	1.23 ± 0.35	–	0.93 ± 0.30

Value is reported as mean ± SD, APDM: APDM Wearable Technologies Inc.

**TABLE 3:** Mean difference of TUG duration from Walking Think mobile application and APDM system, along with 95% limits of agreement (LoA), Pearson’s correlation coefficients (r), and concordance correlation coefficients (r_c_).

TUG duration	Mean difference	95% LoA	*r*	*P*-value	*r*_c_ (95% CI)
TUG	−0.47	−1.83–0.89	0.96	<0.01	0.94 (0.90–0.97)
TUG cognitive	−0.26	−1.56–1.04	0.96	<0.01	0.95 (0.91–0.98)

**TABLE 4:** Mean difference of reaction time from Walking Think mobile application and Multi Choice Reaction Timer derived reaction time, along with 95% limits of agreement (LoA), Pearson’s correlation coefficients (*r*), and concordance correlation coefficients (*r*_c_).

Reaction time	Mean difference	95% LoA	*r*	*P*-value	*r*_c_ (95% CI)
Reaction time	0.30	−0.22–0.82	0.67	<0.001	0.46 (0.27–0.64)

## Discussion

We devised and developed the mobile application called Walking Think that allows easy interaction with users to record the TUG duration during performing walking simultaneously with performing the cognitive dual task, and this mobile application is also able to summarize the results of the data from previous tests for determining the progression or declination of the TUG or TUG-subtraction performances. In addition, Walking Think aimed to allow the clinician to assess reaction time during walking. Findings from this study demonstrated that the Walking Think mobile application is a valid tool to measure the TUG and TUG-subtraction duration. The TUG test was able to distinguish between fallers and non-fallers in community-dwelling older individuals with high sensitivity (80%) and specificity (93%) (10). The TUG-subtraction is helpful for assessing balance while walking. TUG-subtraction has a detrimental effect on functional mobility, and this is demonstrated by the 22–25% longer time required to complete the TUG with an additional secondary task (10). Apart from the traditional paper-based of recording the TUG test, this Walking Think mobile application is appropriate for the screening and follow-up of the TUG and TUG-subtraction performance in routine clinical care.

Nowadays, new technologies, such as wearable sensors or smartphone-embedded inertial sensors, have been created to evaluate the TUG performance of older persons (24). The validity of the unsupervised screening system using sensors (aTUG chair, hip-worn inertial sensor, light barriers, and the Intel^®^ RealSense™ D435 depth image cameras) was examined in a study by Fudickar et al. (25). Their results showed that this system was a valid tool for evaluating TUG, with both light barriers and inertial measurement units demonstrating high correlations with conventional stopwatch measurement (*r* = 0.73 and 0.78, respectively). The usefulness of mobile applications for TUG assessment for various purposes was also reported in previous studies. Bergquist et al. (26) and Madhushri et al. (27) created smartphone applications for the elderly for self-test functional performance evaluation by extracting data from smartphone-embedded inertial sensors such as accelerometers, gyroscopes, and magnetometers. Bergquist et al. (26) developed smartphone applications for physical self-tests including the TUG, tandem stance, and Five Times Sit-to-Stand tests. This study also assessed the usability problems that affected the test’s validity and made adjustments to reduce trial error and update the new instruction. Another study by Madhushri et al. (27) developed mobile health applications including the TUG, 30 s Chair Stand Test, and a 4-stage Balance Test. The TUG application collects and analyzes the incoming signal from the smartphone’s gyroscope and accelerometer sensors, extracting parameters, and displaying the outcomes on the screen. Additionally, it may be feasible to monitor the progress of mobility and balance impairment by analyzing the parameters that were gathered over a long period of time. Also, a mobile application was created with the intention of screening and managing fall risks in the elderly as reported in the study by Taheri-Kharameh et al. (28). This application offers education and training recommendations based on an individual’s level of fall risk among older adults. Moreover, a mobile device application was also able to detect improvement in kinematics and timing during gait and turning components of TUG. According to a study by Koop et al. (29), gait and turning during the TUG can be quantitatively measured using an inertial measuring unit (IMU) on a mobile device. This device was able to distinguish between changes in total time, gait, and turning performances in patients with Parkinson’s disease with on and off-medication conditions.

Apart from the TUG, the Walking Think mobile application was also designed to evaluate the reaction time. Traditionally, psychogeriatric research frequently uses the computerized cognitive battery for assessing the reaction time (30, 31). When compared to a conventional device, a handheld mobile device offers the advantages of being able to alert users to detect vibrations without requiring a visible display and being applicable in a number of settings, such as in a quiet or noisy environment. However, our results showed a moderate correlation between the reaction time detected by the application and the Multi Choice reaction timer. This is probably due to the different stimulus modalities being transduced through different receptive pathways, i.e., auditory neural pathway and somatosensory neural pathway (32). For the mobile application, the tactile stimuli were produced through a vibration of the mobile phone. Once the participants detected a vibration, they pressed their fingers as quickly as possible. While for the Multi Choice Reaction Timer, the auditory stimuli were generated and the participants pressed their fingers as quickly as possible when they detected a noise. Previous studies reported that different modality stimuli influence reaction time. In the context of the man-machine interaction, the study by Ng and Chan (13) examined finger response time to stimuli in the visual, auditory, and tactile modalities. They demonstrated that the response time to auditory stimuli was significantly longer than the response time to tactile stimuli. While the study by Altinsoy (32) showed that reaction times to auditory stimuli were shorter than tactile stimuli. Our findings were in agreement with the study by Altinsoy (32) which the reaction times to auditory stimulus generated by the Multi Choice Reaction Timer was shorter than the tactile stimulus generated by the application. The findings of the validity of the mobile device to evaluate response time in our study were different from the previous study by Burke et al. (33). They reported that information processing *via* simple reaction time with visual stimuli (a green stimulus light) employed on a mobile device was valid and reliable. To assess the validity of mobile devices, the reaction time data were analyzed using a Welch *t*-test to determine if there were any significant differences between traditional and iPad/iTouch. Their results indicated no significant difference in simple reaction time between devices.

### Clinical application

In addition to the traditional use of TUG assessment, this study provided the alternative of using the Walking Think mobile application for recording TUG duration, TUG-dual duration, and reaction time. The TUG and TUG-cognitive duration derived from this mobile application demonstrated excellent correlation in older adults, making them potentially useful in clinical practice.

## Conclusion

The results of this study demonstrate that the Walking Think mobile application is a valid tool for TUG duration, TUG-subtraction duration, and reaction time. The validity of this application for evaluating reaction times may need to be increased by further development.

### Study limitation

Some limitations are noted in this study. Firstly, the participants are all female and have a high function of mobility, therefore, the generalization of results is limited. Secondly, the measurement of reaction time in this study was obtained only in sitting, thus further studies investigating the reaction in various positions are required.

## Data availability statement

The original contributions presented in this study are included in the article/supplementary material, further inquiries can be directed to the corresponding author.

## Ethics statement

The studies involving human participants were reviewed and approved by the Mae Fah Luang University Ethics Committee on Human Research (MFU EC). The patients/participants provided their written informed consent to participate in this study.

## Author contributions

AP conceived and designed the project, the function of procurement, data gathering, analysis, and interpretation of data. RB assisted with the critical revision of the manuscript for important intellectual content. SK and PK assisted with the instrumentation. PS helped in the analysis and interpretation of data. All authors contributed to the article and approved the submitted version.
